# Self-optimizing neural network in the classification of real valued data

**DOI:** 10.7717/peerj-cs.1020

**Published:** 2022-06-28

**Authors:** Alicja Miniak-Górecka, Krzysztof Podlaski, Tomasz Gwizdałła

**Affiliations:** Department of Intelligent Systems, Faculty of Physics and Applied Informatics, University of Lodz, Lodz, Poland

**Keywords:** Classification, Discretization, Machine learning, SONN, SVM

## Abstract

The classification of multi-dimensional patterns is one of the most popular and often most challenging problems of machine learning. That is why some new approaches are being tried, expected to improve existing ones. The article proposes a new technique based on the decision network called self-optimizing neural networks (SONN). The proposed approach works on discretized data. Using a special procedure, we assign a feature vector to each element of the real-valued dataset. Later the feature vectors are analyzed, and decision patterns are created using so-called discriminants. We focus on how these discriminants are used and influence the final classifier prediction. Moreover, we also discuss the influence of the neighborhood topology. In the article, we use three different datasets with different properties. All results obtained by derived methods are compared with those obtained with the well-known support vector machine (SVM) approach. The results prove that the proposed solutions give better results than SVM. We can see that the information obtained from a training set is better generalized, and the final accuracy of the classifier is higher.

## Introduction

It was has been more than 80 years ago since Tryon (1939) published the article, which is currently often considered the first one to consider the problem of grouping data according to some features. These problems were later more precisely formulated and now are well-known as the problems of clustering and classification. In our article, we concentrate on the second one—the classification. The crucial question in the classification process is identifying the relationships between the parameters, which finally leads to the possibility of predicting the belonging to a given class. The typical division of classification procedures considers their belonging to the group of deep or shallow techniques. While deep learning is usually connected with the existence of numerous layers, ultimately forming the structure of an artificial neural network, the methods of shallow learning typically use less extensive structures. We can show here such techniques as k-nearest neighbors (Fix & Hodges, 1951), naive Bayes inference (Kononenko, 1990; Hand & Yu, 2001), random forests (Ho, 1995; Breiman, 1996), or support vector machines (Boser, Guyon & Vapnik, 1992; Cortes & Vapnik, 1995).

A variety of applications for the classification process encompasses many scientific areas. A quick view of the popular databases shows tens of thousands of papers where it is studied. Recently it has become very popular, *e.g.*, in medical applications. We can find different classification techniques in different fields, such as cardiology (Ali et al., 2019), neurology (Deng et al., 2021), or oncology (Murthy & Bethala, 2021). Among other problems studied with the use of classification techniques, we can find anomaly detection in aerospace telemetry (Wu et al., 2020) as well as some form of return to seminal Tryon’s paper—the sentiment analysis deduced from written texts (Wazrah & Alhumoud, 2021) or the sophisticated topic of Fine-Grain Classification, here on the example of malware detection (Fu et al., 2018).

Indeed, the set of several of the most famous and most popular classification techniques mentioned earlier is only a limited selection. The propositions of new methods or new data representations are also numerous, and their authors try to struggle with the problem based on very different assumptions. Among the examples, we can enlist such approaches as the use of hierarchical likelihood in connection with support vector machines applied to the identification of fish species (Caraka et al., 2020) or the Ant Colony Optimization for the recognition of some particular human behavior (Saba et al., 2021). The other interesting approaches are recurrent neural networks with long short-term memory as used for sentiment analysis (Wazrah & Alhumoud, 2021), sparse data approach to pedestrian motion analysis (Chen et al., 2019), or economic prediction (Karasu & Altan, 2022). The other and one of the most interesting solutions is credit rating with hierarchical genetic networks (Pławiak et al., 2020). Certainly, the approaches presented above belong to both classes: deep and shallow learning.

The article presents the classification method, which belongs to the second group (shallow learning) of presented techniques, although called historically after Neural Networks. It is based on ideas presented in the works (Horzyk & Tadeusiewicz, 2004; Horzyk, 2012). The crucial idea is to minimize the size of the neural network by pruning the unnecessary connections, which is called self-optimizing neural networks (SONN). The authors define the mechanism of this approach based on mainly two phases. In the first one, the dataset is discretized, and then the values of so-called discriminants are calculated. Finally, we obtain the system which deterministically classifies the items from the test set.

In the article, we present the modification of the original SONN approach. We propose two different ways how the algorithm incorporates mentioned discriminants. These two versions of the proposed algorithm take into account the ordering of the discriminants while building output from the network. Additionally, we also discuss how the neighborhood topology can influence method results. We use two well-known distance measures, Manhattan and Chebyshev. The proposed method is applied to three different datasets. The first one, the Iris Data Set (Dua & Graff, 2017; Fisher, 1936), is chosen due to its simplicity and popularity. The other two have different properties when considering the number of parameters and the number of classes, but both are often used in similar problems. One of them is the binary problem of Banknote Identification Data Set (Dua & Graff, 2017; Gillich & Lohweg, 2010), the Combined Cycle Power Plant Data Set (CCPP) (Dua & Graff, 2017). These datasets have been used in many publications, for example, in Bandić, Hasičić & Kevrić (2020); Ferencz & Domokos (2020); Rabby et al. (2021); Afzal et al. (2021); Ferdushi et al. (2020); Lohweg et al. (2013); Sang, Dang & Zhao (2021); Li et al. (2021); Gu, Angelov & Prancipe (2018); Tufekci (2014); Kaya, Tufekci & Gurgen (2012). These three datasets allow us to analyze the effectiveness and compare it with other methods. The comparison of results obtained with the proposed method and SVM allows verifying the proposed approach properly. SVM is one of the most recognized methods in the family of the shallow learning techniques and is often used as a reference solution (de Santana et al., 2021; Katoch, Singh & Tiwary, 2022; Vukovic et al., 2022; Al-Azawi, 2022).

It should be mentioned that the proposed method starts with data discretization. It reduces the influence of local variations in data parameters as well as the impact of the uncertainty of the raw data. The discretization process leads to some information loss, for example, when compared with the SVM that works on original floating-point values. However, the results presented in the article show that the proposed method can produce results visibly better than SVM. The uncertainty problems are discussed in many papers, but the authors often use more sophisticated solutions like LSTM networks (Karasu & Altan, 2022; Podlaski et al., 2021). Here we use the shallow learning approach that is much less demanding on the resources. However, in this article, we are not discussing the influence of the method on uncertainty reduction.

## Classification—Self Optimizing Neural Network

This article describes the use of the self-optimizing neural network (SONN). Its structure is related to the adaptation process based on given learning data. The construction process distinguishes the most general and discriminating features of the data. This network can adapt the topology and its weights in the deterministic process (Horzyk & Tadeusiewicz, 2004).

### SONN formalism

The idea of Self-Optimizing Neural Networks (SONN) was introduced in Horzyk & Tadeusiewicz (2004); Horzyk (2012) In this subsection, we show a recapitulation of SONN formalism introduced by Horzyk.

Let $\mathcal{U}$ be a set of the form:

(1) \begin{eqnarray*}\mathcal{U = } \left\{ ({u}^{n},{c}^{n}) \right\} ,\end{eqnarray*}

where ${\mathbf{u}}^{n}=[{u}_{1}^{n},{u}_{2}^{n},\ldots ,{u}_{F}^{n}],$ ${u}_{f}^{n}$ is the value of the *f*-th feature for *n*-th pattern and ${u}_{f}^{n}\in \{ -1,0,1\} $, *n* = 1, 2, …, *N*_*P*_,  *N*_*P*_ is the total number of patterns, *f* = 1, 2, …, *N*_*F*_, *N*_*F*_ is the number of features, *c*^*n*^ is the class for the *n*-th pattern and ${c}^{n}&isin; {\mathcal{N}}_{\mathcal{C}}$, where ${\mathcal{N}}_{\mathcal{C}}$ is the set of classes.

Let ${P}_{f}^{c}$ denote the number of patterns with values greater than 0 in *f*-th features in *c*-th class and ${M}_{f}^{c}$ denote the number of patterns features with values less than 0 as noted

(2) \begin{eqnarray*}{P}_{f}^{c}=\sum _{{u}_{f}^{i}\in \left\{ {u}_{f}^{i}\gt 0,i=1,2,\ldots ,{Q}^{c} \right\} }{u}_{f}^{i},\nonumber\\\displaystyle {M}_{f}^{c}=\sum _{{u}_{f}^{i}\in \left\{ {u}_{f}^{i}\lt 0,i=1,2,\ldots ,{Q}^{c} \right\} }{u}_{f}^{i},\end{eqnarray*}

where *Q*^*c*^ is the number of patterns in the given *c* class.

Having unknown values in a particular feature in the discrete value vector (marked with zero), we can estimate the probability of the occurrence of the feature with a value one or −1. We use the values of this feature in other patterns from the class under consideration. Then, a useful formula is the probabilistic determination of the value of this feature (the middle part of each formula (3)).

(3) \begin{eqnarray*}{x}_{{f}^{+}}^{n}= \left\{ \begin{array}{@{}ll@{}} \displaystyle 1 &\displaystyle {u}_{f}^{n}=1\\ \displaystyle \frac{{P}_{f}^{c}}{{P}_{f}^{c}+{M}_{f}^{c}} &\displaystyle {u}_{f}^{n}=0\\ \displaystyle 0 &\displaystyle {u}_{f}^{n}=-1 \end{array} \right. ,{x}_{{f}^{-}}^{n}= \left\{ \begin{array}{@{}ll@{}} \displaystyle 1 &\displaystyle {u}_{f}^{n}=-1\\ \displaystyle \frac{{M}_{f}^{c}}{{P}_{f}^{c}+{M}_{f}^{c}} &\displaystyle {u}_{f}^{n}=0\\ \displaystyle 0 &\displaystyle {u}_{f}^{n}=1 \end{array} \right. ,\end{eqnarray*}

where ${x}_{{f}^{+}}^{n}$, ${x}_{{f}^{-}}^{n}$ are the approximate values of the indeterminate features. Then the patterns Eq. (2) are of the form

(4) \begin{eqnarray*}{\hat {P}}_{f}^{c}=\sum _{{u}_{f}^{i}\in \left\{ {u}_{f}^{i}\gt 0,i=1,2,\ldots ,{Q}^{c} \right\} }{x}_{{f}^{+}}^{i},{\hat {M}}_{f}^{c}=\sum _{{u}_{f}^{i}\in \left\{ {u}_{f}^{i}\lt 0,i=1,2,\ldots ,{Q}^{c} \right\} }{x}_{{f}^{-}}^{i}.\end{eqnarray*}

Even though SONN allows the use of indefinite values and the special masking procedure has been proposed, we do not deal with such data in our work, therefore

(5) \begin{eqnarray*}{\hat {P}}_{f}^{c}={P}_{f}^{c}{\hat {M}}_{f}^{c}={M}_{f}^{c}\end{eqnarray*}

The described theoretical basis will allow us to understand the method of calculating the coefficient of discrimination (‘Fundamental coefficient of discrimination’).

### Data preparation

Self-optimizing neural networks are adapted to operate on discrete values. Therefore, when working on a certain set of patterns (*i.e.,* elements contained in a data set), we considered a vector of values representing them individually. This vector represents discrete features of a pattern. It has values from the set { − 1, 0, 1}, where −1 means that a feature does not appear in a given pattern, by 0 we denote a feature that is undefined, and 1 means that the pattern has values in a given feature. Regardless, SONN works on discrete feature space, we can use it for real valued datasets like: Banknote Authentication dataset, Iris dataset, and Combined Cycle Power Plant dataset (Dua & Graff, 2017). The real features existing in dataset (*i.e.,* petal length for Iris) we call attributes. We use a special procedure of discretization such datasets. We adjusted each of these sets to use the self-optimizing neural network. For instance, the Iris dataset has four attributes represented as real numbers (*x*, *y*, *z*, *t*). Table 1 shows selected ten patterns from the Iris dataset (the whole dataset has 150 patterns).

**Table 1:** Ten selected patterns from the Iris dataset, described by real value vectors, consist of four attributes (*x*, *y*, *z*, *t*). Each pattern belongs to a specific class.

	iris attributes				
	*real values vector*				
*pattern*	*x*	*y*	*z*	*t*	*class*
*1*	5.1	3.5	1.4	0.2	*0*
*2*	4.9	3	1.4	0.2	*0*
*3*	4.7	3.2	1.3	0.2	*0*
*4*	7	3.2	4.7	1.4	*1*
*5*	6.4	3.2	4.5	1.5	*1*
*6*	6.9	3.1	4.9	1.5	*1*
*7*	5.5	2.3	4	1.3	*1*
*8*	6.3	3.3	6	2.5	*2*
*9*	5.8	2.7	5.1	1.9	*2*
*10*	7.1	3	5.9	2.1	*2*

Each of *p*-th patterns in a dataset is described by a real attributes vector

(6) \begin{eqnarray*}{\mathbf{v}}^{n}=[{v}_{1}^{n},{v}_{2}^{n},\ldots ,{v}_{a}^{n}],\end{eqnarray*}

where 1 ≤ *n* ≤ *N*_*P*_, *N*_*P*_ is the total number of patterns and ${v}_{a}^{n}$ is the value of *a*-th *attribute* for *n*-th pattern, *a* = 1, 2, …, *N*_*A*_, *N*_*A*_ is the total number of attributes.

In our article, we divide the range of variation of each *N*_*A*_ attribute into *r* uniform parts. Split by other division (*e.g.*, clustering) methods are also possible. By uniformly dividing the range of each of the four attributes into three parts, we get three subranges (Table 2). In this way, we obtained a sequence of twelve discrete features representing the pattern (Table 3). Those features we call a discrete feature vector or discrete value vector. We distinguish attributes (real dataset features) from features (pattern discrete features). As was mentioned, if the attribute value of a given pattern is an inappropriate subrange, we put one in the corresponding place of the discrete feature vector, otherwise −1. If we have an attribute with an undefined value, we set the appropriate elements of the feature vector as 0. Described discrete feature vector we denote as

\begin{eqnarray*}{\mathbf{u}}^{n}=[{u}_{1}^{n},{u}_{2}^{n},\ldots ,{u}_{F}^{n}], \end{eqnarray*}

where ${u}_{f}^{n}$ is the value of the *f*-th feature for *n*-th pattern (see 1).

The described discretization procedure can be applied to any real dataset.

**Table 2:** Range of Iris attributes and subranges. Three subranges are formed by uniformly dividing the range of each of the four attributes into three parts.

	range						
	*min*	*max*		subranges			
*x*	4.7	7.1	*x*	[4.7,5.3)	[5.3,5.9)	[5.9,6.5)	[6.5,7.1]
*y*	2.3	3.3	*y*	[2.3,2.6)	[2.6,2.9)	[2.9,3.2)	[3.2,3.5]
*z*	1.3	6	*z*	[1.3,2.475)	[2.475,3.65)	[3.65,4.825)	[4.825,6]
*t*	0.2	2.5	*t*	[0.2,0.775)	[0.775,1.35)	[1.35,1.925)	[1.925,2.5]

**Table 3:** Twelve discrete features represent one pattern. The features have values from the set { − 1, 0, 1}, where −1 means that a feature does not appear in a given pattern, zero indicates a feature that is undefined, and one means that the pattern has values in a given feature.

	*x*			*y*			*z*			*t*		
	[4.4,4.73)	[4.73,5.07)	[5.07,5.4]	[2.9,3.23)	[3.23,3.57)	[3.57,3.9]	[1.3,1.43)	[1.43,1.57)	[1.57,1.7]	[0.1,0.2)	[0.2,0.3)	[0.3,0.4]
	features vector/discrete values vector											
*pattern*	***1***	*2*	*3*	*4*	*5*	*6*	*7*	*8*	*9*	*10*	*11*	*12*	*class*
*1*	−1	−1	1	−1	1	−1	1	−1	−1	−1	1	−1	*0*

### Fundamental coefficient of discrimination

The discrimination coefficient (discriminant) concerns statistical differences in features quantity after their values in classes and the learning dataset. The bigger discriminant, the more important and distinguishing the feature is in view of classification (Horzyk & Tadeusiewicz, 2004).

Discriminants describe how well the feature *f* distinguishes class *c* from other classes, considering their patterns in the learning set.

Horzyk & Tadeusiewicz (2004) proposed the following formula for the discriminants

(7) \begin{eqnarray*}{d}_{f}^{{c}^{+}}= \frac{{\hat {P}}_{f}^{c}}{({N}_{C}-1){Q}^{c}} \sum _{\begin{array}{@{}l@{}} \displaystyle h=1\\ \displaystyle h\not = c \end{array}}^{{N}_{C}} \left( 1- \frac{{\hat {P}}_{f}^{h}}{{Q}^{h}} \right) ,{d}_{f}^{{c}^{-}}= \frac{{\hat {M}}_{f}^{c}}{({N}_{C}-1){Q}^{c}} \sum _{\begin{array}{@{}l@{}} \displaystyle h=1\\ \displaystyle h\not = c \end{array}}^{{N}_{C}} \left( 1- \frac{{\hat {M}}_{f}^{h}}{{Q}^{h}} \right) ,\end{eqnarray*}

(8) \begin{eqnarray*}{d}_{f}=\max \nolimits \{ {d}_{f}^{+},{d}_{f}^{-}\} ,\end{eqnarray*}

where ${d}_{f}^{{c}^{+}}$ and ${d}_{f}^{{c}^{-}}$ mean the coefficient of determination calculated for a given feature of a pattern in the appropriate class, taking into account the values of positive or negative features, respectively. Moreover *c* denotes the class, *c* = 1, 2, …, *N*_*C*_ and *Q*^*c*^ is the number of patterns in the given class *c*. The discrimination coefficients’ values can run across the whole set of real numbers.

### Structure of the network and the weight factor

The structure of the network is related to the described discretization procedure (‘Data preparation’). It was mentioned that this method bases on division into subranges and then maps the real value attributes to the discrete values vector. (see example in Tables 1–3).

Having computed discriminants for each feature of each pattern, we have a matrix representing these values.

The first step in constructing the network was to rank the features of each pattern according to the chosen ordering (see 2.5). Horzyk proposed a certain method of network construction consisting of the iterative addition of successive layers of the network based on features that best distinguish patterns from a given class among all other patterns from other classes (Horzyk & Tadeusiewicz, 2004).

We replaced these concepts with a set of unique (on the training set scale) pairs consisting of the discrete values vector (features) and the corresponding class. Therefore, this pair is of the form:

(9) \begin{eqnarray*}({\mathbf{u}}^{\mathbf{s}},{c}^{s}),\end{eqnarray*}

where **u**^*s*^ is the discrete vector and *c*^*s*^ is the corresponding class. From now on, we will call each such vector a *path*, and all paths will be a set denoted by $\mathcal{S}$. We will call a *neuron* each vector element used to create a *path*.

The resulting structure can be identified with a decision network (which shows similarities to neural networks) or a graph. There are situations where one *path* is associated with many classes and vice versa, that one class is related to several paths.

No matter how the structure was formed, the connections between neurons have a certain weight factor. The coefficient is calculated based on the properly organized network (in our case, identified by the set of *path* s $\mathcal{S}$).

The weight factor for a neuron *j* of a given *path s* is calculated as follows

(10) \begin{eqnarray*}{w}_{j}^{s}={u}_{j} \frac{{d}_{j}^{s}}{\sum _{\begin{array}{@{}l@{}} \displaystyle i\lt =j \end{array}}{d}_{i}^{s}} ,\end{eqnarray*}

where *u*_*j*_ is the value of the feature on the basis of which the neuron *j* is created, ${d}_{j}^{s}$ is the value of the discriminant on the basis of which this neuron is created. Moreover, ${d}_{i}^{s}$ specifies the value of the discriminant on the *path* under consideration $s&isin; \mathcal{S}$, for *i* <  = *j* neurons, *i.e.,* all those neurons (with appropriate discriminants), which were considered before together with the current one. Note that according to the chosen ordering features with appropriate discriminants, there is a change to the denominator (see 10 and 2.5).

### Network response

We tested some modifications of ordering features of each pattern during our work and calculate the weight factor. For further research, we chose two methods of calculating the response from the network for a given pattern.

#### Algorithm version *v*_1_.

In the first one, the order of the features was consistent with the decreasing value of the discriminants, firstly considering the feature set one, and then −1. The chosen ordering features with appropriate discriminants are related to the fact that we consider the value one, *i.e.,* the existence of a given feature, to be more important. The standard formula for calculating the response (the output of a given *path s*) from the network is as follows

(11) \begin{eqnarray*}ou{t}_{s}=f(\sum _{i=0}^{{N}_{F}-1}{w}_{i}^{s}{c}_{i}),\end{eqnarray*}

where ${w}_{i}^{s}$ stands for the weight factor for the given neuron *i* on *path s*,  $s&isin; \mathcal{S}$ and *f* is the activation function.

#### Algorithm version *v*_2_.

In the second approach, we used the decreasing ordering of discriminants. The response from the network for a given pattern is given by

(12) \begin{eqnarray*}ou{t}_{s}=f({d}_{max}^{s}\sum _{i=0}^{{N}_{F}-1}{w}_{i}^{s}{c}_{i}),\end{eqnarray*}

where ${d}_{max}^{s}$ is the highest value of the discriminant on *path s*,  $s&isin; \mathcal{S}$, ${w}_{i}^{s}$ is the weight factor for a given neuron *i* on the *s path* and *f* is the linear function. As we already know (see 2.3) coefficient of discrimination measures the relationship between features and path. High values of the discriminant mean that a given feature is important for a given path (perhaps only for this one) The use of ${d}_{max}^{s}$ may change the relative strength of the individual paths depending on the discriminants. The low value of the discriminants shows that there is little impact. The greater the value, we can assume that it becomes a certain component that allows strengthening the impact of the result for a given *path*.

The *path* introduced in Eq. (9) joins the feature vector with the corresponding class. We want to identify the vector **u**^**s**^ with a hypercube. The well-known method of presenting the multi-dimensional data is its placing in the *N*_*A*_-dimensional space, where *N*_*A*_ is the number of attributes. In such an approach, the feature vector **u**^**s**^ identifies unambiguously one of the *N*_*A*_-dimensional hypercubes, defined, in this space, by the real attributes division into subranges.

**Figure 1:**
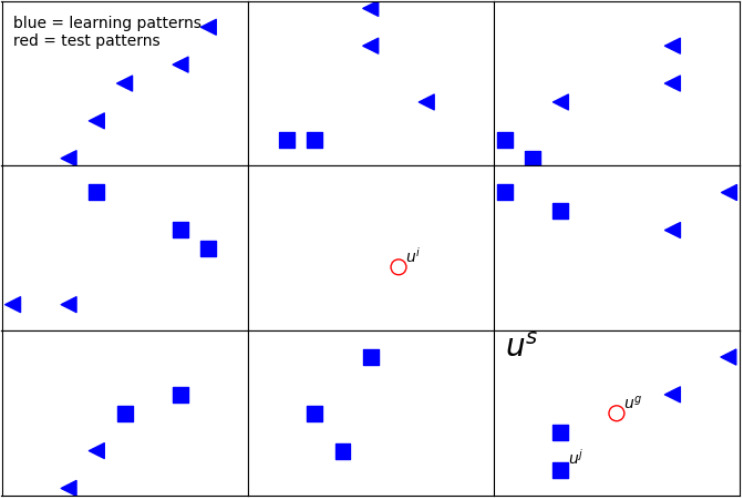
The example of a 2D defined neighborhood for computing the network response for a given pattern *u*^*i*^. The blue points represent the learning patterns (*i.e., u*^*j*^), whereas the red ones (*u*^*i*^, *u*^*g*^) are the test patterns. The shape of the blue markers indicates the distinction between classes. Vector *u*^*s*^ is graphically represented as a rectangle in two-dimensional space. The detailed description of the feature vectors is in Table 4.

For an exemplary illustration, we show Fig. 1 and Table 4. In this figure, blue points are the learning patterns, whereas red ones are the test patterns we want to assign to a certain class. Furthermore, the shape of the blue markers indicates the distinction between classes. For simplicity, in this article, hypercubes in two dimensions are rectangles. The considered **u**^**s**^ is graphically represented as a rectangle in two-dimensional space. As can be noticed, in the rectangle (marked **u**^**s**^), there are filled markers in two shapes. It means they have the same features vector **u**^**s**^, *i.e.,* they belong to the same hypercube. Moreover, there are two *path* with different classes.

When calculating the answer from the network for a certain test pattern, we use a defined neighborhood describing *path* s located at a certain distance from the test pattern and having an impact on the obtained result (Fig. 1). We used two different metrics: Chebyshev and Manhattan. Each of them is responsible for determining which paths contribute to the network’s response.

(13) \begin{eqnarray*}ou{t}_{s}=ou{t}_{s}\ast {2}^{-\mu ({\mathbf{u}}^{\mathbf{i}}-{\mathbf{u}}^{\mathbf{s}})},\end{eqnarray*}

where *μ*_*Manhattan*_ and *μ*_*Chebyshev*_ are the well known metrics to calculate distance between hypercubes, *N*_*A*_ is the number of real attributes, **u**^**i**^ and **u**^**s**^ are the feature vectors form the *i* pattern and the path *s*, respectively.

**Table 4:** Two exemplary patterns are *u*^*i*^ and *u*^*j*^, together with *u*^*s*^ they are the feature vectors representing the *i*th pattern, *j*th pattern and the path *s*. Due to the fact that there are only two real features (*x*, *y*), the considered *u*^*s*^ is graphically represented as a rectangle in two-dimensional space.

	feature vector					
	*x*			*y*		
*pattern*	*1*	*2*	*3*	*4*	*5*	*6*
**u** ^**i**^	−1	1	−1	−1	1	−1
**u** ^**j**^	−1	−1	1	1	−1	−1
**u** ^**s**^	−1	−1	1	1	−1	−1

Regardless of the used modification, the evaluation of the winning class, for each element in the test set, is as follows:

(14) \begin{eqnarray*}ou{t}_{\sigma }=\max _{s\in \mathcal{S}} \left\{ ou{t}_{s} \right\} \text{for}\sigma \in \mathcal{S}.\end{eqnarray*}

The finding *path σ* gives the highest network response, and its class is the winning one.

The important information concerning the method is its computation complexity. It is ruled by Eq. (2), which assumes the analysis of all pattern vectors and all features inside these vectors. We can estimate the complexity of the learning phase as *O*(*N*_*P*_∗*N*_*F*_) where *N*_*P*_ is the number of patterns in the training set and, defined earlier, *N*_*F*_ - the number of features. For the classification process, Eq. (11) gives us direct estimation of single pattern recognition as *O*(*N*_*F*_).

## Experiment and results

In Fig. 1, two exemplary test patterns **u**^**i**^ and **u**^**g**^ (red markers) are shown. One of them (**u**^**i**^) is placed in the rectangle without the training patterns, and the other **u**^**j**^ has in the nearest neighborhood the training patterns. When one or more patterns from the training set are in the same rectangle and belong to different classes, as the answer, to the given test pattern, we choose the class that gives the highest response from our network (see ‘Network response’). In case there is no learning class in a given rectangle, we consider the influence of the neighbors; by taking into account appropriate metrics Eq. (13).

In our work, for research, we focus only on test patterns that do not have learning patterns in their rectangle (like **u**^**i**^ in Fig. 1). Therefore, we use only such test patterns in our results, and they are selected for statistics.

We compare the results of our method with one of the most important classification approaches and the most known method for general application—the SVM method.

### Data

As was mentioned before, we show how our procedure works for the data from the Machine Learning Repository (Dua & Graff, 2017). We choose the Iris dataset, Banknote Authentication dataset and Combined Cycle Power Plant dataset.

The Iris dataset (Dua & Graff, 2017; Fisher, 1936) is one of the most popular collections used for machine learning problems. It is used as the first and simplest dataset to verify clustering methods. This small dataset comprises 150 patterns representing three uniform classes (Iris Setosa, Iris Versicolor, and Iris Virginica). Each pattern is represented by a vector of four attributes (sepal length, sepal width, petal length, and petal width).

The next is the Banknote Authentication dataset (Dua & Graff, 2017; Gillich & Lohweg, 2010), which was prepared according to a special procedure. The banknotes images were analyzed using the wavelet transform, and several characteristics were collected from such transformed data. The set comprises 1,372 patterns belonging to two classes(binary classes). The values of five attributes describe patterns, and the statistical moments like variance, kurtosis or skewness, and also the entropy of the image. There are many references in the literature to the classification issue related to these data, *i.e.,* Kumar & Nagamani (2018) and Shrivas & Gupta (2017).

The most complicated of investigated datasets is the Combined Cycle Power Plant dataset (CCPP) (Dua & Graff, 2017; Tufekci, 2014; Kaya, Tufekci & Gurgen, 2012). It contains 9,568 patterns gained from a combined-cycle power plant over six years (2006–2011). While collecting, the power plant was set to work with a full load. Patterns are described by attributes: hourly average ambient variables temperature (T), ambient pressure (AP), relative humidity (RH), and exhaust vacuum (V) to predict the net hourly electrical energy output (EP) of the plant. As we can see, all data have floating-point values. Contrary to the above datasets (CCPP), we do not have an explicit class division. The authors of this dataset do not define the number of classes, but we do it through our actions. Using the recursive k-means tool (Miniak-Gorecka, Podlaski & Gwizdalla, submitted), we divide the response set (EP) into three classes. There are many references to the classification issue related to these data in the literature *i.e.,* (Saleel, 2021; Santarisi & Faouri, 2021; Alketbi et al., 2020; Siidiqui et al., 2021).

In Table 5, the datasets based on which we perform the research are listed briefly. In the visualization (see ‘Results’), we used the labeling from the first column. The first three rows contain the information about original datasets from Machine Learning Repository (Dua & Graff, 2017). The remaining *CCPP1*, *CCPP2* were created from the original *CCPP* by reducing the number of elements and using specific selection procedures. The resulting datasets contain 450 elements selected as follows:

**Table 5:** Datasets. Iris, banknote authentication, and combined-cycle power plant are the original datasets from the Machine Learning Repository (Dua & Graff, 2017). The remaining CCPP1 and CCPP2 are created from the original CCPP with a different, specific selection of its elements.

marking	Dataset	nr of instances	nr of attributes
*A*	Iris	150	3
*B*	Banknote Authentication	1372	5
*C*	Combined Cycle Power Plant (CCPP)	9568	4
*D*	CCPP1	450	4
*E*	CCPP2	450	4

CCPP1: –elements are selected randomly from the entire *CCPP*,
CCPP2: –each of three classes is represented uniformly by selected 150 random instances.

We are aware that different selection methods may affect the results obtained; however, as we mentioned, we try to omit the ordering of the dataset.

**Figure 2:**
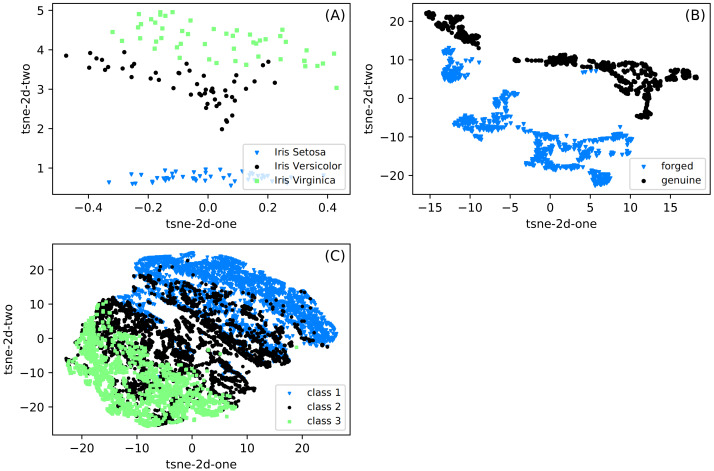
(A–C) Two-dimensional projections of datasets used in classification obtained with the t-SNE method (Hinton & Roweis, 2002). The images shows clearly the possibility of separation of particular classes for the Iris and Banknote datasets while for CCPP it is impossible.

The differences between the datasets used in experiments are presented in Fig. 2. We show the data projection onto a two-dimensional space with the t-SNE method (Hinton & Roweis, 2002). The t-distributed stochastic neighbor embedding (t-SNE) is based on the minimization of Kulback-Leibler divergence. The divergence is calculated between two distributions in the original and two-dimensional target space. As shown in this figure, the distances are usually calculated with the Euclidean metric, but arbitrary metrics can be used. We can observe that classes are well separated for *Iris* and *Banknote* datasets. It is impossible for the *CCPP* dataset to separate classes, especially patterns belonging to class 2, covering a whole range of working parameters. It suggests that for this dataset, the prediction can be most challenging.

### Experiment setup

In the further descriptions of tests and the discussion of the obtained results, we use the terminology presented in Table 6. The differences between two approaches (*algorithm versions*) considered is clarified in ‘Network response’, where the self-optimizing neural network is described. The division specifies the number of {5, 6, …, 15} uniform subranges for each real attributes (Tables 1–3). When calculating the answer from the network for a certain test pattern, we use defined neighborhood pointing *path* s located at a certain distance from the test pattern that has an impact on the obtained result (‘Network response’ and Fig. 1). We use two different *metrics*: Chebyshev and Manhattan. They are responsible for determining which *path* s contribute to the network’s best response. The next term *ratio* specify what percentage of all patterns in dataset enters the training (80, 70, 60, 50) and test (20, 30, 40, 50) set, respectively.

**Figure 3:**
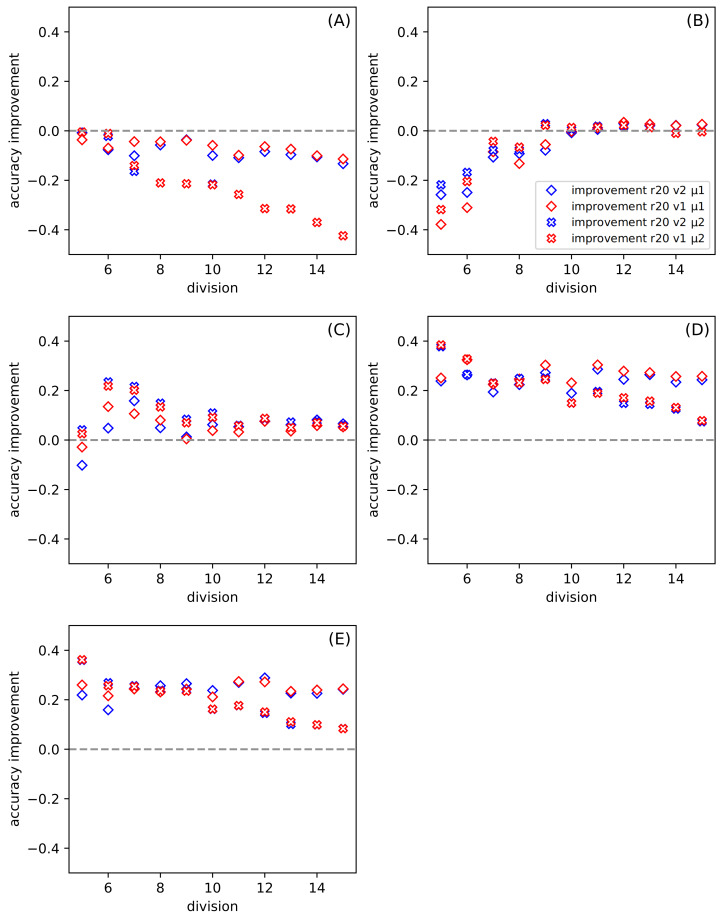
The dependence of the improvement on the division *d* among all datasets for ratio *r* = 20, for the set of parameters: version *v* and metric *μ* (see Table 6). (A–E) present results for one of the datasets described in Table 5. The positive value of the improvement indicates that compared method gives better results than the classic SVM.

**Table 6:** Set of parameters. The differences between two approaches (algorithm versions) is clarified in subsection 2.5. The division specifies the number of {5, 6, …, 15} uniform subranges for each real attributes (Tables 1–3). Two metrics: Chebyshev and Manhattan determine which paths contribute to the network’s best response. The term ratio specify what percentage of all patterns in dataset is in the training (80, 70, 60, 50) and test (20, 30, 40, 50) set. Therefore a tuple (called the set of parameters) (*v*, *d*, *μ*, *r*) i.e., (*v*1, *d*5, *μ*1, *r*20) gives us the unambiguous description of the process.

marking	description
*algorithm version (v)*	see section 2.5 for versions *v*1 and *v*2
*division (d)*	division of the parameter range on uniform subranges in number from 5 to 15
*metric* (*μ*)	*μ*_1_ - *Chebyshev*, *μ*_2_ - *Manhattan*
*ratio (r)*	percentage 20, 30, 40, 50 of all patterns in test set
*average* (avg_SONN_)	average of good answers for 30 tests each with a given configuration of parameters
*average* (avg_SV M_)	average of good answers for 30 tests
*improvement*	(avg_SONN_ − avg_SV M_)

All presented results of the recognition of test patterns are based only on those test patterns that do not have an equivalent among the training patterns. It means that in our approach, no **u**^**s**^, $s&isin; \mathcal{S}$ from *path* s can efficiently classify a given test pattern (see (9) and Fig. 1). By the good answers we mean those answers that match the expected output (expected class). In addition, for each of the possible configurations of parameters (version, division, metric, ratio) we perform thirty tests. We denote as avg_SONN_ the average of good SONN answers for thirty of those tests for each given configuration of parameters. As avg_SVM_ we mark the average of good SVM answers for those thirty tests (applied parameters do not affect the operation of the SVM method). The scale of improvement determines how much our method is better (worse) than the considered classic SVM and it is calculated as follows

(avg_SONN_ − avg_SVM_).

We also point out that the classic SVM works on real values (attributes), while our method is based on their discrete forms (in the form of discrete vectors) and our method has the possibility of setting a combination of parameter values (version, division, metric, ratio) to improve the network response.

All figures included have a uniform format for presenting data. The shapes of markers (diamonds and crosses) correspond to different *metrics*. The color (red and blue) indicates the version of approach. Filling the shape gives us a distinction in terms of ratio. For all pictures, we use the naming entered in the Table 6.

### Results

In our experiments, we focused only on test patterns that do not have learning patterns in their hypercube (like **u**^**i**^ in Fig. 1). We establish that more than five of these test patterns must be in the set. For each configuration of parameters (version, division, metric, ratio) we perform thirty tests. The results presented in this section are the average results of the tests performed.

Figure 3 shows the dependence of the improvement on the division for all datasets. The *ratio* is set to twenty. In this case, the variable parameters are *version* and *metric*. Each of Figs. 3A–3E presents results for a separate dataset (Table 5). The positive value of the improvement indicates that compared method gives better results than the classic SVM. In our work, we use the python version of SVM(with the following parameters: kernel =’poly’, *C* = 1, gamma =’scale’, degree =3) (Pedregosa et al., 2011), and we provide the results of this tool so that we do not have to go into other research on this method. The higher the value of improvement, the more suitable our method is. Figures 3A and 3B show how the tested methods behave in the case of well-known and small datasets Iris and Banknote Authentication, respectively. We note that the SVM method works well for Iris. Currently, it is possible to find research carried out with the use of Iris dataset in various sources, in which, after numerous changes, even one hundred percent effectiveness was obtained. The SVM version is also specially modified for this dataset there. It should be noted that SVM uses more information than the proposed SONN method. The discretization procedure is responsible for this difference; the real-valued attributes have more information than the discrete value vector. With the increase of the division parameter, the values of the improvement become negative, and the difference between SVM and our results increases. SVM seems to be more suitable in that case. For the Banknote dataset, while increasing the partition density, we get the highest number of empty hypercubes. The average number of paths per cube decreases as the split is denser. For test patterns falling into empty hypercubes, we calculate the network response based on the responses of adjacent cubes. The influence of adjacent hypercubes increases when patterns are more sparsely distributed. For partition density above ten, our results and SVM results are practically the same. Although, for both so far discussed datasets, the results are unsatisfactory. We can observe the differences in the plots (Figs. 3A and 3B) presented. For one of the datasets (Iris) the performance of our method is visibly worse than SVM, but for Banknote we can notice the similarity of the results for both techniques. We want to test our method using more demanding data; that is why we also use the *CCPP* set containing almost ten thousand patterns. With the increase of the complexity of the studied data (Figs. 3C–3E), our method looks more efficient. All four Figs. 3C–3E show the results for *CCPP*. The results in Fig. 3C present the different behavior than the former ones (Figs. 3A and 3B). To better understand the behavior of average improvement, we also present its standard deviation (Table 7). We can see that with the growth of division parameter, it does not only increase the improvement but also its deviation decrease. It suggests that the proposed methods with both metrics give statistically better results than SVM. In order to assess the dependence of total datasets size, we create two subsets of *CCPP* dataset. These subsets preserve the complexity of the total CCPP dataset and are even more sparse distributed. For these subsets, we select the number of patterns settled between sizes of Iris and Banknote datasets. Figures 3D–3E are created, for the needs of research, excluding the ordering source data (Table 5). An interesting effect (especially Fig. 3E) is that for small sets we get a significant improvement. For some divisions, our method turns out to be much better than SVM used on the same data. Even for denser divisions (more than ten subranges), our method seems to be statistically better than SVM. As it is shown in Table 7 the average improvement exceeds the standard deviation for the higher value of the division. The Manhattan metric gives better results for small values of division, but when we go towards tighter divisions, it is surpassed by Chebyshev metric. Almost always, except for the crosses in Fig. 3E (symbolized Manhattan metric), our results are better. The amount of improvement is significant because it oscillates between 0.2 − 0.4, and it is more closely related to the metric than to the version.

**Table 7:** Average improvement and its standard deviation obtained in thirty tests for each configuration of parameters on CCPP dataset. The visualization of the average improvement value is shown in Fig. 3C.

	*versionv*, *metric *μ* and ratior* = 20							
	(*v*1, *μ*1)		(*v*1, *μ*2)		(*v*2, *μ*1)		(*v*2, *μ*2)	
*divisiond*	*improvement*	*deviation*	*improvement*	*deviation*	*improvement*	*deviation*	*improvement*	*deviation*
*5*	−0.1020	0.2546	0.0412	0.1506	−0.0285	0.2166	0.0253	0.1824
*6*	0.0482	0.3143	0.2349	0.2153	0.1350	0.2975	0.2181	0.2739
*7*	0.1583	0.1825	0.2165	0.1625	0.1064	0.1619	0.2015	0.1573
*8*	0.0494	0.1172	0.1490	0.0950	0.0806	0.0735	0.1337	0.0786
*9*	0.0127	0.0847	0.0839	0.0570	0.0044	0.0626	0.0696	0.0569
*10*	0.0620	0.0712	0.1093	0.0673	0.0380	0.0683	0.0913	0.0585
*11*	0.0532	0.0358	0.0596	0.0417	0.0323	0.0409	0.0578	0.0445
*12*	0.0766	0.0280	0.0859	0.0345	0.0755	0.0327	0.0877	0.0327
*13*	0.0610	0.0321	0.0726	0.0227	0.0359	0.0345	0.0505	0.0342
*14*	0.0812	0.0269	0.0764	0.0224	0.0581	0.0293	0.0698	0.0277
*15*	0.0656	0.0224	0.0612	0.0227	0.0528	0.0268	0.0553	0.0244

**Figure 4:**
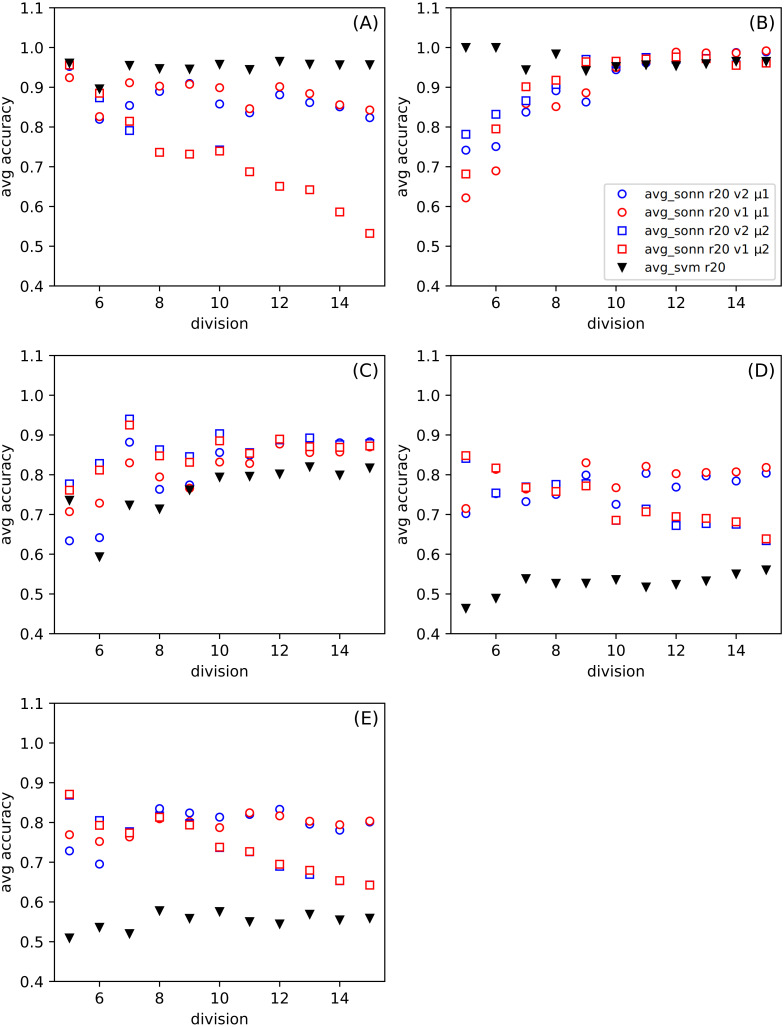
(A–E) The comparison of the average accuracy for SONN and for SVM. The average is taken over thirty independent divisions into training and test sets with a ratio *r* = 20. The parameters (version *v* and metric *μ*) for the particular dependence of SONN results on the division type are presented in the legend. This kind of plot allows for analyzing the absolute result for a particular set of parameters and, therefore, finding a source of dependence shown in Fig. 3.

In Fig. 3, we show the differences between the average of good responses from thirty tests for SONN and SVM, whereas in Fig. 4, we present absolute accuracy for both methods on selected datasets and configuration of parameters. These results allow us to determine whether the result is the effect of SONN method improvement or deterioration of both methods in the entire range of parameters and for all datasets. Figures 4A and 4B (related to relatively simple Iris and Banknote Authentication datasets) indicate that, regardless of the version and metric selection, SVM gives better results. Considering the more numerous, more complicated datasets, we indicate that our method gives significantly better results than the classic SVM approach for the datasets Figs. 4D–4E. Also, we show that the average number of good answers among tests achieves the best results for the set Fig. 4E).

**Figure 5:**
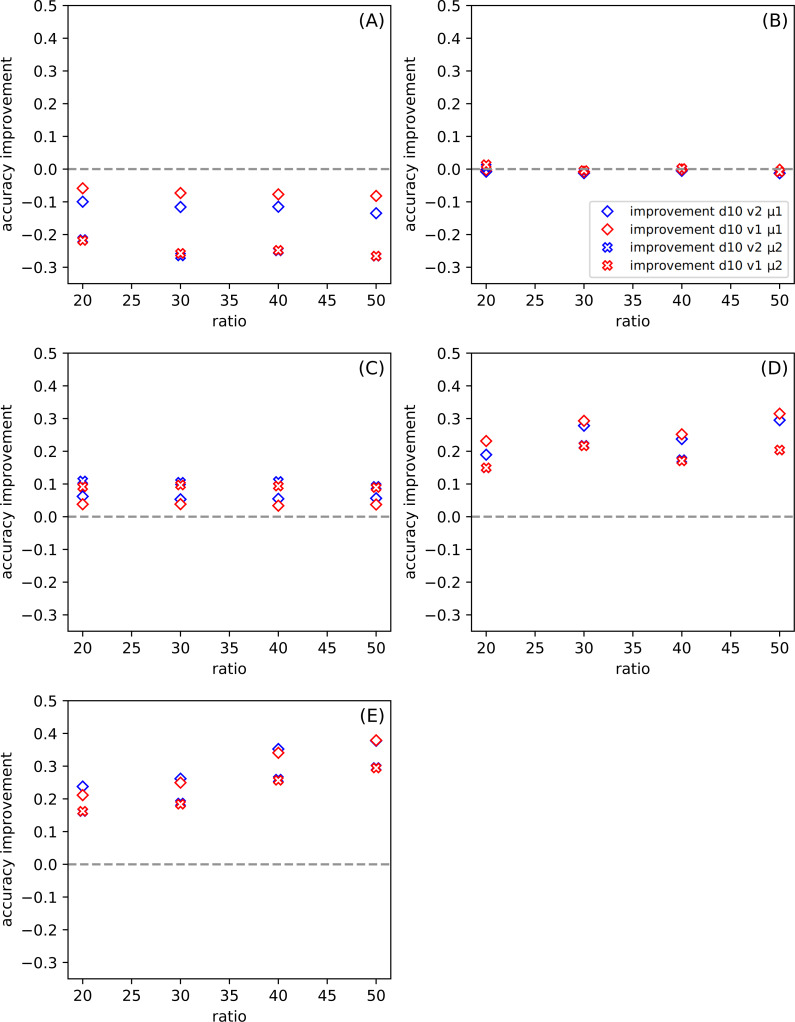
(A–E) Dependence of the improvement on the ratio *r* among all datasets (see Table 5) for division *d* = 10 (see other divisions in Fig. 3), for the set of parameters: version *v* and metric *μ* (described in the legend).

Choosing only one from the attributes subranges (*d*10) from all presented in Figs. 3 and 4, in Fig. 5 we show the dependence the improvement of the ratio among all datasets for the set of parameters: *version* and *metric*. Presenting the test results in this form, we also observe an improvement for the aforementioned sets (Figs. 5D and 5E).

**Figure 6:**
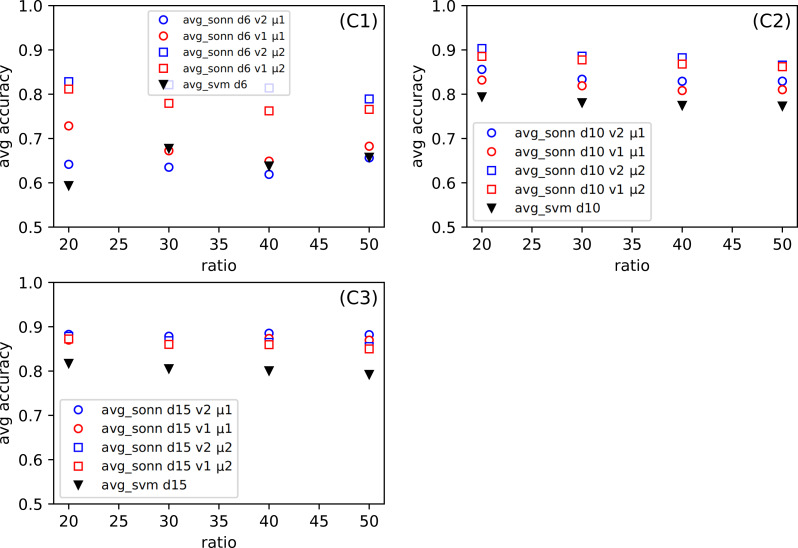
(C1–C3) The comparison of the average accuracy for SONN and for SVM. The average is taken over thirty independent CCPP (C) tests for SONN on the whole range of ratio *r*. The parameters are: division (only *d*6, *d*10, *d*15), version (*v*1, *v*2) and metric (*μ*1, *μ*2).

One of our goals is to show that the method we created can be successfully used for various types of datasets. Even though we examine several datasets (among them were the simple, standard ones), we want to focus on the more complicated ones, namely the Combined Cycle Power Plant Dataset. Figure 6C, for CCPP shows the dependence the comparison of the average response from thirty tests for SONN and for SVM approach. The parameters for SONN are: the whole range of ratio, division (only *d*6, *d*10, *d*15), version and metric. In Fig. 4C we only observed the test results for ratio 20, now we have a broader look at the behavior of CCPP. In the case of dividing the range of parameters variability into six uniform subranges (Fig. 6C1), in the case of two sets of parameters ((*v*1, *μ*1), (*v*2, *μ*1)) we observe a decline in the correctness of our method. For the remaining Figs. 6C2 and 7C3, the approach we propose gives much better results.

**Figure 7:**
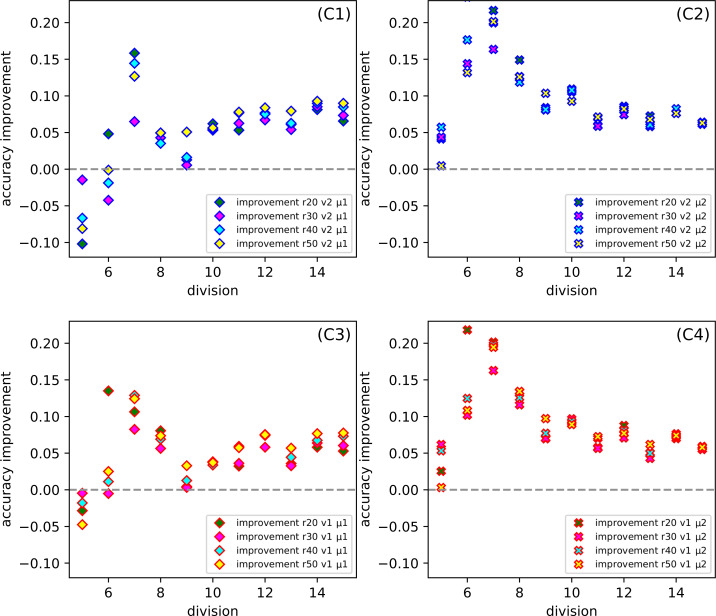
(C1–C4) The dependence of the improvement on the division *d* of CCPP (C) for the set of parameters: the whole range of ration *r*, version (*v*1, *v*2) and metric (*μ*1, *μ*2).

Figure 7 gives us a look at how the *CCPP* behaves in the case of different partitions of the parameters into uniform subranges. In more detail, it shows the dependence the improvement of the *division* for the set of parameters: ratio, version and metric. Comparing all Figs. 7C1–7C4 at the same time, it seems that for *division d*7 we get the best results, while the *division d*5 gives the worst effectiveness. However, on closer inspection, we can also indicate that the selection of the *μ*2 *metric* gives a satisfactory result for more than one subrange division.

## Conclusion

In the article, we presented the applicability of the self-optimizing neural network in classifying real-valued data. When summarizing the approach, we have to point out some properties which significantly influence the computational procedure.

First of all, unlike in many other methods, we have to prepare data by some form of preprocessing; discretization is the crucial process. The discretization parameters: the method of division and the number of intervals into which the entire variable area is divided can significantly affect results. Therefore, we carefully studied these dependencies. It should also be mentioned that we do not use the typical multi-dimensional clustering techniques here to distinguish the subspaces of whole variable space into subareas with similar properties. Indeed, the clustering process can be used only for each dimension separately. The approach presented in the article made it possible to avoid some basic, difficult-to-answer questions concerning, *e.g.*, the number of clusters or the metric used to define the distance between particular instances.

The proposed method is, for historical reasons, related to neural networks. In fact, we do not observe the typical learning process concerning the strength of particular bonds in the system of defined topology. The learning process is related to analyzing the strength of particular branches defined by the vectors obtained by the discretization, performed with the described earlier scheme. We introduced several modifications to the original method. In particular, they are designed to weaken the importance of two factors: less decisive values of variables (see Eq. (12)) and the role of the more distant neighborhood (see Eq. (13)).

In order to test our method, we selected three datasets that differ from each other in dimensionalities and number of classes. We started from the small and well-known Iris dataset with four parameters and three classes, through Banknote Identification (five parameters, binary) to CCPP (four parameters, without direct division into classes).

We can concentrate on different issues when estimating the prediction accuracy related to multi-dimensional classification problems. The typical way is to show the results for the whole test set, but we decided to present them differently. By dividing every variable into some number of intervals, we create a multi-dimensional grid of hypercubes. The distribution of points from the training as well as testing sets is not uniform among these hypercubes. So, we can identify the points for which the decision path is not determined after analyzing the training set. We think that the analysis of results concentrated on these points better reproduces the ability of the system to generalize the data during the learning process.

For the data mentioned above, our classification results were compared with those obtained with the Support Vector Machine. SVM is a popular and widely accepted technique for solving classification problems. The comparison confirms the high quality of results obtained using our modification of the SONN method. Although the details may depend on some parameters used in the procedure (version, metric), we can generally say that the accuracy obtained using SONN is higher than for SVM. Only for the most straightforward dataset, IRIS, the results are different. For other ones, the effects of SONN can outperform SVM even by 20–30%. Considering the comparison of absolute values, the improvement still is about a few percent for the worse cases. It can be noticed that for these cases, SONN improves the SVM result being already close to 100% (see Fig. 4). When taking into account that we concentrate on the points which are outside the determined paths, we can expect the better behavior of the proposed method for the outliers.

When going into some general remarks commenting on the efficiency of the SONN method, we can mention several features. SONN seems to behave better with the increase in the density of divisions. Except for the Iris dataset, the accuracy increased significantly when dividing all the variables into smaller intervals. It may be related to the observation that with such an increase, we create a higher number of paths with better-defined classes, so the influence on the neighborhood is also better defined. The comparison between datasets shows that we get better results with the increasing complexity of the dataset. This effect is visible when looking, *e.g.*, at the t-SNE diagrams. The first impression about discretization leads to the impoverishment of information. Despite it, however, the localization of cases in particular discretized areas can improve classification accuracy.

The method seems to be promising, and as the main directions of further development, we can enlist: the effect of the method on uncertainty reduction and the reconstruction of experimental data (Podlaski et al., 2021).

## Additional Information and Declarations

### Data Availability

The following information was supplied regarding data availability: The datasets, code and results obtained in the modelling process are available at http://ksi.fis.uni.lodz.pl/works_repo/sonn_and_datasets/.
